# Processes and Outcomes of Care for Soft Tissue Sarcoma of the Extremities

**DOI:** 10.1080/13577140220127521

**Published:** 2002-03

**Authors:** Lawrence Paszat, Brian O'Sullivan, Robert Bell, Vivien Bramwell, Patti Groome, William Mackillop, Emma Bartfay, Eric Holowaty

**Affiliations:** 1 Radiation Oncology Research Unit Department of Oncology Queen's University Kingston Canada; 2 Institute for Clinical Evaluative Sciences Toronto Canada; 3 Department of Radiation Oncology University of Toronto Toronto Canada; 4 Department of Surgery University of Toronto Toronto Canada; 5 Department of Medicine University of Western Ontario London Canada; 6 Cancer Surveillance Unit Cancer Care Ontario and University of Toronto Canada; 7 Toronto Sunnybrook Regional Cancer Centre 2075 Bayview Avenue Toronto Ontario M4N 3M5 Canada

## Abstract

*Purpose:* A population-based cohort study of soft tissue sarcoma of the extremities (STSE) in Ontario, Canada was conducted using linked administrative databases.

*Methods and materials:* Electronic administrative databases were linked from the Ontario Cancer Registry, the Canadian Institute for Health Information, and Radiation Oncology Research Unit database of radiation therapy (RT) records.

*Results:* The definitive surgery was amputation for 6.0%, resection for 60.9%, biopsy for 7.5%; the remainder had no surgical record. Adjuvant RT was administered to 40.2% of cases. Among cases initially treated by surgical resection, 2.0% later underwent amputation and 9.5% underwent further resection during follow-up. The adjusted odds ratio (OR) for amputation as definitive surgery was 2.3 (1.19, 4.45) in eastern Ontario relative to Toronto. The likelihood of adjuvant RT among those not registered at a cancer centre within 3 months of diagnosis was decreased (OR = 0.20 (95% CI (0.13, 0.30)) relative to those registered. The adjusted relative risk of amputation at any time following diagnosis was 3.48 (95% CI (1.63, 7.46)) among cases not attending a cancer centre. The adjusted relative risk of death was 1.4; 95% CI (1.1, 1.7) among those not attending a cancer centre.

*Conclusions:* Cases not seen at a multidisciplinary cancer centre within 3 months following diagnosis of STSE have an increased relative risk for amputation at any time, and for death due to any cause. Many hypotheses for further study are suggested by the results of this analysis.


*Sarcoma* (2002) **6**, 19–26

Taylor & Francis healthsciences

**ORIGINAL ARTICLE**

### Processes and outcomes of care for soft tissue sarcoma of the extremities

LAWRENCE PASZAT[1,2,3], BRIAN O'SULLIVAN[3], ROBERT BELL[4], VIVIEN BRAMWELL[5], PATTI GROOME[1], WILLIAM MACKILLOP[1], EMMA BARTFAY[1] & ERIC HOLOWATY[6]

[1]*Radiation Oncology Research Unit, Department of Oncology, Queen's University, Kingston, Canada,* [2]*Institute for Clinical Evaluative Sciences, Toronto, Canada,* [3]*Department of Radiation Oncology, University of Toronto, Canada,* [4]*Department of Surgery, University of Toronto, Canada,* [5]*Department of Medicine, University of Western Ontario, London, Canada &* [6]*Cancer Surveillance Unit, Cancer Care Ontario and University of Toronto, Canada*

**Abstract**

*Purpose:* A population-based cohort study of soft tissue sarcoma of the extremities (STSE) in Ontario, Canada was conducted using linked administrative databases.

*Methods and materials:* Electronic administrative databases were linked from the Ontario Cancer Registry, the Canadian Institute for Health Information, and Radiation Oncology Research Unit database of radiation therapy (RT) records.

*Results:* The definitive surgery was amputation for 6.0%, resection for 60.9%, biopsy for 7.5%; the remainder had no surgical record. Adjuvant RT was administered to 40.2% of cases. Among cases initially treated by surgical resection, 2.0% later underwent amputation and 9.5% underwent further resection during follow-up. The adjusted odds ratio (OR) for amputation as definitive surgery was 2.3 (1.19, 4.45) in eastern Ontario relative to Toronto. The likelihood of adjuvant RT among those not registered at a cancer centre within 3 months of diagnosis was decreased (OR = 0.20 (95% CI (0.13, 0.30)) relative to those registered. The adjusted relative risk of amputation at any time following diagnosis was 3.48 (95% CI (1.63, 7.46)) among cases not attending a cancer centre. The adjusted relative risk of death was 1.4; 95% CI (1.1, 1.7) among those not attending a cancer centre.

*Conclusions:* Cases not seen at a multidisciplinary cancer centre within 3 months following diagnosis of STSE have an increased relative risk for amputation at any time, and for death due to any cause. Many hypotheses for further study are suggested by the results of this analysis.

**Key words:** *soft tissue sarcoma of extremity, processes of care, outcomes of care, population-based health services research*

### Introduction

Although soft tissue sarcoma of the extremities (STSE) among adults comprises less than 1% of incident cancers, the burden of suffering for cases of STSE is great, because of disfigurement, disability, and mortality.

Most surgeons and oncologists rarely treat patients with STSE and do not develop expertise in its diagnosis and treatment. Processes for the diagnosis and treatment of STSE are very complex. There are international recommendations for the care of STSE patients that mandate consultation with subspecialized surgical, radiation, and medical oncologists, prior to definitive treatment.[1–5] The Society for Surgical Oncology and the National Comprehensive Cancer Network have published consensus-based guidelines on some aspects of the care of STSE.[6,7] However, the proportion of STSE cases receiving such care is unknown. Such care is recommended in oncology in general,[8] and has been associated with improved outcomes in population-based studies of other malignancies.[9,10]

The goal of treatment for STSE is to avoid mutilation and/or disability whenever possible, while offering each patient the highest possibility of curing the disease.[11–14] In reports of RCTs and institutional case series, there is evidence that correctly performed treatments directed at limb preservation, with function acceptable to patients, are effective in appropriate cases.[12,13,15] Procedures must be correctly selected and performed, for highest likelihood of histopathologically clear resection margins, limb preservation, and good function.[6,16–19]

Population-based studies of STSE have indicated much lower rates of referral to expert services than

Correspondence to: Lawrence Paszat, BA, MD, MS, FRCPC, Toronto Sunnybrook Regional Cancer Centre, 2075 Bayview Avenue, Toronto, Ontario, Canada M4N 3M5. Tel.: +1-416-480-5847; Fax: +1-416-480-6002; E-mail: lawrence.paszat@tsrcc.on.ca

1357–714X print/1369–1643 online/02/010019–08 © 2002 Taylor & Francis Ltd
DOI: 10.1080/1357714022012752 1

predicted.[2,3,20] Some biopsies are performed incorrectly,[21] and histopathology reporting may be incorrect or inadequately reported, and expert review may be omitted. Omission or incorrect selection or interpretation of diagnostic imaging are common.[2]

If inexpert surgical procedures are performed in the first instance,[2,6,16–19] there may be a higher probability of unsatisfactory surgical resection margins, with a higher rate of local failure, and a higher number of surgical procedures performed on STSE. Clasby *et al.*[2] concluded that best available evidence for treatment had not been applied. Inappropriate surgery had been performed, including unnecessary amputation. A total of 67.1% of resection specimens contained unsatisfactory resection margins. Adjuvant RT had been omitted despite being indicated by the extent of disease. Clasby *et al.*[2] deemed that overall only 60% of cases had been treated adequately.

This paper presents a population-based cohort study of STSE in Ontario. The objectives of the study were: (1) to describe STSE case volumes of hospitals and cancer centres, in order to express the magnitude of institutional experience in the treatment of cases of STSE as a surrogate measure of specialized expertise; (2) to describe the proportion of cases admitted to hospitals with the largest experience in the treatment of cases of STSE and the proportion of cases of STSE that attend a multidisciplinary cancer centre (as a surrogate measure of multidisciplinary care); and (3) to describe the treatment of newly diagnosed STSE and clinical outcomes, in relation to institutional case volume.

### Methods

### *Study design*

The study design is a population-based cohort of all cases of STSE diagnosed in Ontario between 1 January 1987 and 31 December 1996, who were aged 17 and older at the time of diagnosis. The study is based on linked electronic administrative health services databases originally intended for other purposes: the Ontario Cancer Registry (OCR), hospital files from the Canadian Institute for Health Information (CIHI), and radiotherapy (RT) files from all RT facilities in Ontario, compiled by the Radiation Oncology Research Unit (RORU), Queen's University at Kingston, Ontario.

### *Data sources and variables*

The OCR files included the following variables: ICD diagnosis, ICD-O histology code, diagnosis date, age at diagnosis, Ministry of Health residence code, postal code, numeric cancer centre label, and cancer centre chart number. The OCR ascertains cases on the basis of data from the following records originally created for other purposes: (1) registrations at cancer centres; (2) pathology reports received directly from acute care hospitals; (3) hospital discharge abstracts received from the Canadian Institute for Health Information (CIHI); and (4) death certificate diagnoses.[22]

Electronic hospital discharge abstracts from CIHI contain the admission date, discharge date, and major treatment procedures. The validity and reliability of electronic reports of cancer-directed surgery has been described by Holowaty *et al.*[23] Surgical treatment directed at the primary STSE was categorized from the Canadian Classification of Procedures codes contained in the CIHI files as 'amputation', 'resection', 'biopsy' or 'other'.

Electronic RT records from all cancer centres in Ontario were compiled, processed and analyzed by RORU, which linked these files to the OCR case files. Only 1% of cancer patients have a record of RT in original charts but none in the electronic file.[24] Records of adjuvant RT were selected by the codes describing the anatomic region treated.

These linked data do not contain information about the stage or grade of STSE at diagnosis, physician characteristics, the completeness or reliability of diagnostic imaging or pathology reporting, diagnostic delay, the use of chemotherapy, or direct histological or radiological ascertainment of local recurrence and/ or distant metastasis of STSE.

### *Process of care variables*

#### *'Case volumes of hospitals and cancer centres'*

STSE case volumes during the study period were calculated for hospitals of first admission, for hospitals performing maximal surgical procedures within 8 months of diagnosis, and for cancer centres, in order to describe the STSE experience of the institutions as a surrogate for specialized expertise in the treatment of STSE. Cancer centres are distinguished from hospitals, which are inpatient and surgical facilities; the regional cancer centres in Ontario are chiefly ambulatory facilities which are the sole providers of radiation oncology services, the major providers of medical oncology services, and the chief foci of multidisciplinary care encompassing surgical, radiation, and medical oncology consultation and other supportive oncological disciplines.

#### *'Attendance at multidisciplinary cancer centre within 3 months following diagnosis'*

All permanent residents of Ontario are entitled to consultation and treatment without charge. Cases attending a multidisciplinary cancer centre within 3 months of diagnosis were identified from OCR records, which contain the date of enrollment at cancer centres.

*'Region of residence'*

The province of Ontario was divided into regions defined as the areas served by each multidisciplinary cancer centre. The STSE cases residing in each census subdivision were assigned to the region of whichever cancer centre was most frequently attended by residents of the census subdivision.[24,24]

*'Initial treatment period'*

Definitive surgery and adjuvant RT performed within 8 months of the diagnosis of STSE were distinguished from those performed subsequently, in order to identify initial treatment. The 8-month window was selected in order to capture the cases that had a preoperative or postoperative combination of surgery and RT.

*'Definitive surgery for primary STSE'*

A hierarchy of surgical procedures was established, and only the highest ranking procedure during the first 8 months following diagnosis was selected as the definitive surgery; amputation ranked highest, followed by resection, followed by biopsy (lesser procedures are not considered in the study).

*Analysis of the processes of care*

The case volumes of: (1) the hospital of first admission, and (2) the case volumes of the hospital where the maximal surgery directed at the primary STSE was performed, were computed. The proportion of cases of STSE attending a cancer centre within 3 months of diagnosis was computed. These proportions and frequencies were categorized to express STSE case volume.

*Rates of definitive surgery and adjuvant RT*

Univariate and bivariate analysis of the rates of definitive surgery and of adjuvant RT were performed. Chi-square tests were performed and $p$ values on the statistic were reported. Multivariate logistic regression analyses were performed to assess: (1) the likelihood of amputation as the definitive surgery, and (2) the likelihood of adjuvant RT.

*Outcome variables*

Amputation at any time between diagnosis and last contact or date of death was an outcome variable. Amputation or resection directed at the primary anatomic site of STSE at any time during follow-up after definitive surgery were considered to be surrogate outcome measures of progressive or recurrent STSE at the primary site. Death due to any cause was an outcome.

*Outcome analysis*

Univariate and bivariate analyses using the life table method were performed, to assess the time to amputation at any time (censoring all deaths), and overall survival. Multivariate Cox proportional hazards regression analysis was used to model: (1) the risk of amputation conditional on survival (censoring cases from the analysis at the time of death due to any cause), and (2) the risk of death from any cause, controlling for age, gender, histology, region of residence, year of diagnosis, case volume of first hospital and whether or not a case attended a cancer center within 3 months after diagnosis.

### Results

*Description of study population*

The OCR contains records of 1467 cases of STSE diagnosed between 1 January 1987 and 31 December 1996. ICD 171.2 (upper limb and shoulder girdle) comprised 23.6% of cases, ICD 171.3 (lower limb) 58.5%, and ICD 171.6 (buttock and pelvic girdle) 17.9% of the 1467 cases. Cases of STSE increased from 244 in 1987–1988 to 335 in 1995–1996 (Table 2).

*Analysis of processes of care*

*STSE case volumes of hospitals*

Patients were initially admitted at any one of 147 hospitals; 135 hospitals admitted fewer than 20 new cases of STSE during the 10 years, 11 admitted between 20 and 50 cases, and one hospital admitted more than 50 cases. The percent of cases initially admitted (1) at a hospital with < 20 cases of STSE during 1987–1996 was 47.0%, (2) at a hospital with 20–50 cases 20.7%, and (3) at a hospital with > 50 cases 18.6%. There was no hospital record in the CIHI electronic files for 13.7% of cases.

*STSE case volumes of cancer centres*

The percentage of STSE cases attending a cancer centre was 56.7% within 90 days of diagnosis. Six cancer centres received < 100 cases of STSE during the study period; two centres received between 100 and 200 cases and one centre received > 200 cases.

*Hospitals performing definitive surgery*

Patients underwent definitive surgery at one of 139 hospitals. One hundred and thirty of these hospitals performed definitive surgery on fewer than 20 cases in total during the 10 years; seven performed definitive surgery on between 20 and 50 cases during the 10 years; two performed definitive surgery on more than 50 cases during the 10 years.

There was only a small shift of cases from hospitals of first admission with smaller case volumes to hospitals with larger case volumes for the maximal surgical procedure: 45.5% had maximal surgery at a hospital with < 20 cases (compared to 47% of first admissions), 13.5% at a hospital with 20–50 cases (compared to 18.6% of first admissions), and 27.3% at a hospital with > 50 cases (compared to 13.7% of first admissions). These are percentages of the entire study population in each case; they do not sum to 100%, or sum to equal total percentages, because some cases had no admission, and some cases with an admission had no surgery.

The proportion of cases undergoing one surgical procedure directed at the primary STSE within 8 months after diagnosis was 43.4%; 23.2% underwent two procedures; 7.8% underwent three or more procedures; 25.6% did not undergo any procedure.

The proportion of cases undergoing definitive surgery (Table 1) increased with (1) increasing STSE case volume of the hospital of first admission ($p<0.0001$), (2) increased among those registered at cancer centres within 3 months of diagnosis ($p < 0.0001$), (3) varied among regions of residence ($p < 0.0001$), but (4) did not vary by year of diagnosis ($p = 0.37$).

The percentage of cases undergoing amputation: (1) varied among the regions of Ontario ($p = 0.05$), (2) decreased with increasing STSE case volume of the hospital of first admission ($p = 0.04$) and (3) decreased with attendance at a cancer centre within 3 months of diagnosis ($p = 0.08$) (Table 1). The percentage undergoing amputation did not vary signficantly by year of diagnosis ($p = 0.20$).

### *Rates of adjuvant RT*

The rates of RT increased with: (1) increasing STSE case load of the hospital of first admission ($p < 0.0001$), and (2) increasing attendance rates at a cancer centre within 3 months of diagnosis ($p < 0.0001$) (Table 2). Variation among the regions of Ontario was not significant ($p$=0.51).

Among the cases with amputation as definitive surgery, 21/88 (23.9%) had a record of preoperative adjuvant RT. Among cases with resection as definitive surgery, 59/893 (6.6%) had a record of preoperative adjuvant RT and 390/893 (43.7%) had a record of postoperative adjuvant RT. Among cases having had biopsy only, 45/110 (40.9%) had a record of RT following biopsy. Among cases with no record of a surgical procedure, 75/376 (20.0%) had a record of RT directed at the primary site.

### *Multivariate analyses of treatment utilization*

Multivariate logistic regression analysis (Table 3) demonstrated that the odds ratio (OR) for amputation, adjusted for age, gender, histology, and anatomic site of STSE was elevated in one region (adjusted OR = 2.3; 95% CI (1.1, 7.4)) relative to the Toronto region, but did not vary among the other regions, or among years of diagnosis, STSE case volumes of the hospital of first admission, or by attendance status at a cancer centre within 3 months of diagnosis.

Multivariate logistic regression analysis of adjuvant RT (Table 3) produced ORs for RT, adjusted for age, gender, histology and anatomic subsites, which were increased among those cases first admitted at hospitals with the largest STSE case volume relative to those with the smallest volume (adjusted OR=2.4; 95% CI (1.6, 3.7)), and decreased for cases not attending a cancer centre within 3 months of diagnosis (adjusted OR = 0.2; 95% CI (0.1, 0.3)) relative to those cases who attended. The odds ratios for RT did not vary region of residence, or year of diagnosis.

**Table 1.** *Description of definitive surgery and adjuvant RT, according to clinical variables*

| Variable | Amputation | Resection or biopsy | No procedure | Total | RT directed at primary site |
|---|---|---|---|---|---|
| Age at diagnosis | | | | | |
| 17–29 | 7 (5.6%) | 92 (73.0%) | 27 (21.4%) | 126 | 38 (30.2%) |
| 30–49 | 29 (7.6%) | 278(72.8%) | 75 (19.6%) | 382 | 167 (43.7%) |
| 50–69 | 30 (5.9%) | 349(68.7%) | 129(25.4%) | 508 | 211 (41.5%) |
| 70–79 | 11 (3.9%) | 190(67.4%) | 81 (28.7%) | 282 | 128 (45.4%) |
| > = 80 | 11 (6.5%) | 94 (55.6%) | 64 (37.9%) | 169 | 46 (27.2%) |
| Gender | | | | | |
| Female | 36 (5.4%) | 452(67.4%) | 183(27.3%) | 671 | 274 (40.8%) |
| Male | 52 (3.5%) | 551(69.2%) | 193(24.3%) | 796 | 316 (39.7%) |
| Histology | | | | | |
| Malignant fibrous histiocytoma | 16 (4.7%) | 249(73.7%) | 73 (21.6%) | 338 | 169 (50.0%) |
| Fibrosarcoma | 4 (8.2%) | 31 (63.3%) | 14 (28.6%) | 49 | 24 (49.0%) |
| Liposarcoma | 3 (1.0%) | 248(84.4%) | 43 (14.6%) | 294 | 152 (51.7%) |
| Leiomyosarcoma | 8 (4.7%) | 103(59.9%) | 61 (35.5%) | 172 | 58(33.7%) |
| Rhabdomyosarcoma | 3 (6.7%) | 32 (71.1%) | 10 (22.2%) | 45 | 22 (48.9%) |
| Synovial sarcoma | 15 (21.4%) | 49 (70.0%) | 6 (8.6%) | 70 | 38 (54.3%) |
| Angiosarcoma | 7 (11.3%) | 35 (56.5%) | 20 (32.3%) | 62 | 20 (32.3%) |
| Sarcoma NOS | 18 (12.8%) | 84 (59.6%) | 39 (27.7%) | 141 | 52 (36.9%) |
| Other sarcoma | 10 (5.7%) | 110(62.9%) | 55 (31.4%) | 175 | 54 (30.9%) |
| Clinical diagnosis | 4 (3.3%) | 62 (51.2%) | 55 (45.5%) | 121 | 1 (0.8%) |

**Table 2.** *Description of definitive surgery and adjuvant RT, according to process of care variables*

| Variable | Amputation | Resection or biopsy | No procedure | Total | RT directed at primary site |
|---|---|---|---|---|---|
| Region | | | | | |
| Ottawa | 17 (11.8%) | 91 (63.2%) | 36 (25.0%) | 144 | 55 (38.2%) |
| Toronto | 34 (5.6%) | 440 (71.8%) | 139 (22.7%) | 613 | 262 (42.7%) |
| Hamilton | 8 (3.8%) | 157 (73.7%) | 48 (22.5%) | 213 | 77 (36.2%) |
| Kingston | 3 (4.0%) | 55 (73.3%) | 17 (22.7%) | 75 | 33 (44.0%) |
| London | 14 (7.8%) | 128 (71.5%) | 37 (20.7%) | 179 | 82 (45.8%) |
| NW Ontario | 4 (11.1%) | 20 (55.6%) | 12 (33.3%) | 36 | 13 (36.1%) |
| Windsor | 3 (4.8%) | 45 (72.6%) | 14 (22.6%) | 62 | 29 (46.8%) |
| NE Ontario | 5 (5.4%) | 64 (68.8%) | 24 (25.8%) | 93 | 39 (41.9%) |
| Missing | | | | 52 | |
| Year of diagnosis | | | | | |
| 1987–1988 | 20 (8.2%) | 163 (66.8%) | 61 (25.0%) | 244 | 85 (34.8%) |
| 1989–1990 | 20 (7.7%) | 166 (64.1%) | 73 (28.2%) | 259 | 94 (36.3%) |
| 1991–1992 | 17 (5.7%) | 194 (65.5%) | 85 (28.7%) | 296 | 116 (39.2%) |
| 1993–1994 | 15 (4.5%) | 237 (71.2%) | 81 (24.3%) | 333 | 145 (43.5%) |
| 1995–1996 | 16 (4.8%) | 243 (72.5%) | 76 (22.7%) | 335 | 150 (44.8%) |
| Case volume of first hospital | | | | | |
| < 20 cases | 44 (6.4%) | 528 (76.6%) | 117 (17.0%) | 689 | 275 (40.1%) |
| 20–50 cases | 30 (9.9%) | 230 (75.7%) | 44 (14.5%) | 304 | 150 (49.3%) |
| > 50 cases | 14 (5.1%) | 245 (89.7%) | 14 (5.2%) | 273 | 165 (60.8%) |
| No admission | | | | 201 | |
| Registration at centre > = 3 months | | | | | |
| Yes | 58 (65.9%) | 634 (63.2%) | 140 (25.6%) | 832 | 542 (65.1%) |
| No | 30 (34.1%) | 369 (36.8%) | 236 (74.4%) | 635 | 48 (7.6%) |

**Table 3.** *Odds ratios for amputation as definitive surgery, and odds ratios for adjuvant RT, by process of care variables, simultaneously adjusted for clinical variables*

| Variable | Odds ratio for amputation as definitive surgery | Odds ratio for RT directed at the primary site |
|---|---|---|
| Region | | |
| Ottawa | **2.3 (1.2, 4.5)** | 0.9 (0.5, 1.5) |
| Toronto | 1.0 | 1.0 |
| Hamilton | 0.6 (0.3, 1.3) | 0.7 (0.4, 1.0) |
| Kingston | 0.5 (0.1, 1.8) | 1.4 (0.7, 2.8) |
| London | 1.5 (0.7, 3.0) | 0.9 (0.6, 1.5) |
| NW Ontario | 1.1 (0.4, 4.3) | 0.7 (0.3, 1.9) |
| Windsor | 0.9 (0.3, 3.4) | 1.2 (0.6, 2.6) |
| NE Ontario | 0.7 (0.2, 2.2) | 1.1 (0.6, 2.0) |
| Year of diagnosis | | |
| 1987–1988 | 1.7 (0.8, 3.5) | 0.7 (0.5, 1.1) |
| 1989–1990 | 1.8 (0.9, 3.7) | 0.7 (0.5, 1.0) |
| 1991–1992 | 1.3 (0.6, 2.6) | 0.7 (0.5, 1.0) |
| 1993–1994 | 0.9 (0.4, 1.9) | 1.0 (0.7, 1.3) |
| 1995–1996 | 1.0 | 1.0 |
| Case volume of first hospital | | |
| < 20 cases | 1.0 | 1.0 |
| 20–50 cases | 1.5 (0.9, 2.6) | 1.1 (0.9, 1.9) |
| > = 50 cases | 0.7 (0.4, 1.7) | **2.4 (1.6, 3.7)** |
| Registration at cancer centre within 3 months | | |
| Yes | 1.0 | 1.0 |
| No | 1.5 (0.7, 3.0) | **0.2 (0.1, 0.3)** |

*Outcomes analysis*

*Surrogate measures of uncontrolled or recurrent STSE at primary anatomic site*

The electronic data do not contain information about histological confirmation of local recurrence of STSE; however, records of surgery subsequently performed after the initial treatment period are present for 14.2% of cases. Among the 893 cases with surgical resection within 8 months following diagnosis, 18 (2.0%) subsequently underwent amputation and 85 (9.5%) underwent another resection. Among the 110 cases with biopsy within 8 months, four (3.6%) cases subsequently underwent amputation and five (4.5%) cases resection. Among the 376 cases without a record of surgical procedure within 8 months following diagnosis, 7/376 (1.8%) subsequently underwent amputation, and 39/376 (10.4%) resection.

*Analysis of time to amputation*

Analysis of time to amputation (at any time between the date of diagnosis and last follow-up) by the life table method, censoring all deaths, demonstrated a difference among the anatomic subsites (log-rank test $p = 0.002$; Wilcoxon test $p=0.003$).

Multivariate analysis of the time to amputation, using Cox proportional hazards regression (Table 4), censoring deaths due to any cause demonstrated that the relative risk (RR) for amputation, adjusted for age, gender, histology and anatomic subsites, was 3.48 (95% CI (1.63, 7.46)) for those not attending a cancer centre within 3 months, but did not vary among the regions of residence, years of diagnosis, or hospital categories according to STSE case volume.

*Survival analysis*

Overall survival for the entire population was 64.0% at 5 years; cause-specific survival was 80.0%. Overall survival did not vary by region of residence, or case volume of first hospital, but varied by year of diagnosis and by attendance status at a regional cancer centre.

Multivariate Cox proportional hazards regression analysis of overall survival demonstrated a relative risk for death due to any cause, adjusted for age, gender, histology, and anatomic subsite, which was elevated (1) for all years of diagnosis relative to 1995–1996 (Table 4), and (2) for cases not attending a cancer centre within 3 months of diagnosis (RR = 1.4; 95% CI (1.1, 1.7). The adjusted RR for death did not vary among the regions of Ontario relative to Toronto, or according to the STSE case volume of the hospitals of first admission.

### Discussion

*STSE case volumes of hospitals and cancer centres*

For 45% of cases, definitive surgery was performed at a hospital treating < = 2 new cases per year. Only 13.6% of cases admitted to low case volume hospitals were referred to larger case volume hospitals for surgery. A total of 9.4% of cases residing in regions with low case volume cancer centres was referred to centres with larger case volume. The percentage of all cases of STSE attending a multidisciplinary cancer centre within 3 months of diagnosis was 56.7%. Most cancer centres treated fewer than 100 new cases of STSE during the study period.

These observations indicate that recommendations in the clinical literature for expert multidiscplinary care are not implemented for many cases.[1–5,8–10] We hypothesize that many patients may be receiving treatment for STSE at institutions with insufficient case volumes to develop or maintain specialized

**Table 4.** *The relative risk of amputation at any time, and the relative risk of death, by process of care variables, simultaneously adjusted for clinical variables*

| Variable | Relative risk of amputation at any time | Relative risk of death due to any cause |
|---|---|---|
| Region of residence | | |
| Ottawa | 1.7 (0.7, 4.2) | 0.7 (0.5, 1.0) |
| Toronto | 1.0 | 1.0 |
| Hamilton | 0.9 (0.3, 2.2) | 0.8 (0.6, 1.0) |
| Kingston | 0.5 (0.1, 2.5) | 0.7 (0.5, 1.2) |
| London | 1.4 (0.6, 3.6) | 0.7 (0.5, 1.0) |
| NW Ontario | 0.7 (0.1, 6.0) | 1.6 (1.0, 2.6) |
| Windsor | 1.5 (0.4, 5.7) | 0.9 (0.6, 1.4) |
| NE Ontario | 0.7 (0.2, 2.4) | 0.8 (0.5, 1.2) |
| Year of diagnosis | | |
| 1987–1988 | 2.5 (0.7, 9.27) | **1.9 (1.3, 2.7)** |
| 1989–1990 | 3.6 (1.0, 13.1) | **1.6 (1.1, 2.4)** |
| 1991–1992 | 2.0 (0.5, 7.3) | **1.7 (1.2, 2.5)** |
| 1993–1994 | 1.1 (0.3, 4.8) | **1.7 (1.1, 2.5)** |
| 1995–1996 | 1.0 | 1.0 |
| Case volume of first hospital | | |
| < 20 cases | 1.00 | 1.00 |
| 20–50 cases | 1.1 (0.6, 2.7) | 1.1 (0.8, 1.3) |
| > 50 cases | 0.7 (0.3, 1.7) | 0.8 (0.6, 1.1) |
| Registration at cancer centre within 3 months | | |
| Yes | 1.0 | 1.0 |
| No | **3.5 (1.6, 7.5)** | **1.4 (1.1, 1.7)** |

expertise. We hypothesize that many patients treated only in low case volume hospitals and cancer centres do not receive expert multidisciplinary care for STSE.

*Cases without record of surgery or RT*

Cases without record of surgery or adjuvant RT likely include: (1) cases diagnosed with detectable metastatic disease, (2) cases whose comorbid illnesses prevented definitive treatment or whose prognoses from comorbid illness rendered treatment of STSE unnecessary, (3) cases who indeed received surgery and/or RT directed at the primary site of STSE but who lacked an electronic record of the procedures, and (4) cases of very small Stage Ia STSE who underwent a minor excision in a doctor's office and had no hospital procedure and did not require RT.

*Surrogate outcome measures of local recurrence of primary STSE*

The surrogate outcome measure of local recurrence of STSE following primary treatment is the percentage of cases initially treated by surgical resection subsequently undergoing amputations or further resections. A total of 2.0% underwent amputation and 9.5% underwent further resection; this combined percentage (11.5%) is higher than the rate of local recurrence reported in the best single-institution cases series studies.[15] We recall that Gustafson reported local recurrence rates 2.4 times higher among cases of STSE not referred to specialized sarcoma units.[3,4] Population-based STSE outcomes are not optimized in most jurisdictions from which they have been reported.

*Constraints on interpretation of these administrative databases*

Interpretation of these results is constrained by the absence of information on stage including grade of STSE, physician characteristics, diagnostic imaging, pathology reporting, adjuvant chemotherapy, or local recurrence or metastasis of STSE. Because of the absent covariates, it is not possible to compare the care received by these cases to the practice guidelines of the Society of Surgical Oncology[6] or the National Comprehensive Care Network.[7]

Treatment selection biases obviously confound the examination of associations between processes of care and outcomes; therefore, we have avoided making outcome comparisons on the basis of treatment for STSE. Overall survival was positively associated with attendance at a cancer centre within 3 months of diagnosis, but not with region of residence or with case volume of the hospital; this may reflect a referral bias if patients with more advanced STSE or more serious comorbid illness are less likely to be referred to a cancer centre. It is not possible to assess survival benefit from cancer centre attendance with the available covariates.

*Importance of population-based study of processes and outcomes of care*

The study of the processes and outcomes of care on a population basis is important, despite the absence of certain variables from electronic databases. Patients enrolled within clinical trials for their treatment often have better outcomes than patients treated outside of clinical trials. They are rigorously selected to be comparable, and their physicians, treating institutions, and treatment processes are subject to stringent guidelines.[26] The treatment benefits seen in randomized clinical trials may be diminished when the treatment is implemented in the general population, because the spectrum of patients treated will be broader, and there will be a broader range of treatment processes and physician and institutional expertise outside of the context of a randomized clinical trial.[27,28]

Population-based studies are necessary to reveal the processes of care in the general population and to determine aspects of the processes that require improvement. Such studies will never be able to adjust for unknown treatment selection variables or stage, but are useful for the generation of hypotheses for further study.

*Sources of problems in the processes of care for STSE*

Problems with the processes of care for STSE may relate to practitioner and/or institutional inexperience and to failure to disseminate knowledge and guidelines about the care of STSE. The Society for Surgical Oncology has published consensus-based surgical guidelines,[6] and the National Comprehensive Cancer Network has published consensus-based guidelines for a range of diagnostic and therapeutic scenarios in soft tissue sarcoma.[7]

### Conclusion

Multivariate time to failure analyses have shown that cases not attending any cancer centre within 3 months following diagnosis have a higher relative risk of amputation at any time, and a higher relative risk of death from any cause.

### Acknowledgements

Dr Paszat and Dr Groome are career scientists of the Ministry of Health of Ontario. The radiation oncology departments of Ontario provided the radiation therapy data. This work was supported in part by an operating grant from Cancer Care Ontario to the Radiation Oncology Research Unit, and by a cancer outcomes grant from Cancer Care Ontario.

### References

1. Rydholm A. Centralization of soft tissue sarcoma. The Southern Sweden experience. *Acta Orthop Scand* 1997; 68: 4–8.
2. Clasby R, Tilling K, Smith MA, Fletcher CDM. Variable management of soft tissue sarcoma: regional audit with implications for specialist care. *Br J Surg* 1997; 84: 1692–6.
3. Gustafson P, Dreinhofer KE, Rydholm A. Soft tissue sarcoma should be treated at a tumor centre. *Acta Orthop Scand* 1994; 65: 47–50.
4. Gustafson P. Soft tissue sarcoma epidemiology and prognosis in 508 patients. *Acta Orthop Scand Suppl* 1994; 259: 1–31.
5. Wiklund T, Huuhtanen R, Blomqvist C, *et al.* The importance of a multidisciplinary group in the treatment of soft tissue sarcomas. *Eur J Cancer* 1996; 32A: 269–73.
6. Pollock RE, Brennan MF, Lawrence W. Soft tissue sarcoma surgical practice guidelines. *Oncology* 1997; 11: 1327–2.
7. National Comprehensive Cancer Network. Sarcoma Practice Guidelines. *Oncology* 1998; 12: 183–218.
8. Selby P, Gillis C, Haward R. Benefits from specialised cancer care. *Lancet* 1996; 348: 313–8.
9. Danjoux CE, Jenkin RDT, McLaughlin J, *et al.* Childhood medulloblastoma in Ontario, 1977–1987: population-based results. *Med Pediatr Oncol* 1999; 26: 1–9.
10. Hillner BE, Smith TJ, Desch CE. Hospital and physician volume or specialization and outcomes in cancer treatment: importance in quality of cancer care. *J Clin Oncol* 2000; 18: 2327–40.
11. Wilson AN, Davis A, Bell RS, *et al.* Local control of soft tissue sarcoma of the extremity: the experience of a multidisciplinary sarcoma group with definitive surgery and radiotherapy. *Eur J Cancer* 1994; 30A: 746–51.
12. Catton C, Swallow CJ, O'Sullivan B. Approaches to local salvage of soft tissue sarcoma after primary site failure. *Semin Radiat Oncol* 1999; 9: 378–88.
13. Catton C, Davis A, Bell RS, *et al.* Soft tissue sarcoma of the extremity. Limb salvage after failure of combined conservative therapy. *Radiother Oncol* 1996; 41: 209–14.
14. LeVay J, O'Sullivan B, Catton C, *et al.* Outcome and prognostic factors in soft tissue sarcoma in the adult. *Int J Radiat Oncol Biol Phys* 1993; 27: 1091–9.
15. O'Sullivan B, Wylie J, Catton C, *et al.* The Local Management of Soft Tissue Sarcoma. *Semin Radiat Oncol* 1999; 9: 328–48.
16. Heslin MJ, Woodruff J, Brennan MF. Prognostic significance of a positive microscopic margin in high-risk extremity soft-tissue sarcoma: implications for management. *J Clin Oncol* 1996; 14: 473–8.
17. Bell RS, O'Sullivan B, Liu FF, *et al.* The surgical margin in soft-tissue sarcoma. *J Bone Joint Surg Am* 1989; 71A: 370–5.
18. Rydholm A. Surgical margins for soft tissue sarcoma. *Acta Orthop Scand* 1997; 68: 81–5.
19. Pisters PWT, Woodruff J, Brennan MF. Prognostic significance of a positive microscopic margin in high-risk extremity soft tissue sarcoma: implications for management. *J Clin Oncol* 1996; 14: 1679–89.
20. Gaffney EF, Dervan PA, McCabe MM, *et al.* Soft tissue and visceral sarcomas in Irish patients. *Irish J Med Sci* 1994; 163: 240–5.
21. Mankin HJ, Mankin CJ, Simon MA. The hazards of the biopsy, revisited. *J Bone Joint Surg Am* 1996; 78a: 656–3.
22. Clarke EA, Marrett LD, Krieger N. Cancer registration in Ontario: a computer approach. In: Jensen OM, Parkin DM, Maclennan R, *et al.* eds. *Cancer registration: principles and methods*. Lyon, France: Internation Agency for Research on Cancer, 1991; 246–57.
23. Holowaty EJ, Morovan E, Lee G, *et al.* *A reabstraction study to to estimate the completeness and accuracy of data elements in the Ontario Cancer Registry*. A report to Health Canada, HC contract H4078-3-CO98/01-SS, 1996.
24. Mackillop WJ, Groome PA, Zhang-Salomons J, *et al.* Does a centralized radiotherapy system provide adequate access to care? *J Clin Oncol* 1997; 15: 1261–7.
25. Mackillop WJ, Zhang-Salomons J, Groome PA, *et al.* Socioeconomic status and cancer survival in Ontario. *J Clin Oncol* 1997; 15: 1680–9.
26. Begg CB. Engstsrom PF. Eligibility and extrapolation in cancer clinical trials. *J Clin Oncol* 1987; 5: 962–8.
27. Yusuf S, Collins R, Peto R. Why do we need some large, simple randomized trials? *Stat Med* 1984; 3: 409–20.
28. Layde PM, Broste SK, Desbiens N. Generalizability of clinical studies conducted at tertiary care medical centers: a population-based analysis. *J Clin Epidemiol* 1996; 49: 835–41.
